# Effect of Bovine Colostrum on Canine Immune Health

**DOI:** 10.3390/ani15020185

**Published:** 2025-01-11

**Authors:** Ping Yu, Ebenezer Satyaraj

**Affiliations:** Nestlé Purina Research, One Checkerboard Square, St. Louis, MO 63164, USA

**Keywords:** bovine colostrum, nutrients, dogs, immune system, immunomodulatory, vaccination, immunosenescence

## Abstract

Colostrum, the first fluid secreted by the mammary glands of mammalian mothers, contains essential nutrients for the health and survival of newborns. Bovine colostrum (BC) is notable for its high concentrations of bioactive components, such as immunoglobulins and lactoferrin. Despite dogs being the world’s most popular companion animals, there is limited research on their immune systems compared to humans. This summary aims to consolidate published studies that explore the immune benefits of BC, focusing specifically on its implications for dogs.

## Simple Summary

Dogs are the most popular pet in the world and ensuring their long and healthy lives is essential. A robust immune system is crucial for their well-being. One promising approach is using bovine colostrum (BC), rich in immunoglobulins and essential nutrients. This review consolidates information on BC components that support immune health. By examining studies across different life stages of dogs, we aim to highlight BC’s role in enhancing immune function in our canine companions.

## 1. Introduction

Colostrum is the first form of milk produced by mammals after birth, offering a distinct nutrient profile compared to mature milk. It provides essential components like proteins, fats, carbohydrates, and bioactive compounds, including immunoglobulins (Igs), cytokines, lactoferrin, and oligosaccharides, which play key roles in supporting immune function [1]. While the composition of colostrum varies across species, bovine colostrum (BC) is particularly abundant and can be safely utilized across different life stages for human and animal health [1]. BC’s bioactive compounds make it an excellent functional ingredient for immune support, and its widespread availability from the dairy industry opens opportunities for its use in foods, supplements, and animal feed [2]. While different colostrum suppliers have different, sometimes proprietary production processes, the methodologies for quality control such as analyzing products and maximizing shelf life, etc., have been widely standardized in the dairy industry. Costa A and colleagues summarized the progress and proposed the collaboration between the scientific community and stakeholders of dairy chain [3]. Instead of overviewing the field, the current manuscript only highlights BC’s immune-related nutrients, the unique aspects of the canine immune system, and exploring its benefits for canine health.

## 2. Bovine Colostrum Composition

### 2.1. Proteins

Bovine colostrum (BC) contains approximately 15% protein by weight, compared to just 3% protein in mature milk [4]. This protein is present in both soluble and insoluble forms, known as whey protein and caseins, respectively. Both protein components offer significant nutritional and bioactive properties [4].

#### 2.1.1. Whey Protein

Whey protein, a collection of water-soluble proteins found in bovine colostrum, is rich in essential amino acids, making it beneficial for both humans and pets. Among these amino acids, branched-chain amino acids (BCAAs)—including valine, leucine, and isoleucine—are particularly important. BCAAs play a critical role in protein synthesis and muscle repair, making them a key component in many bodybuilding supplements. These amino acids also contribute to energy production and immune support, further enhancing the functional benefits of whey protein.

In an ex vivo model, valine has been shown to influence the function of monocyte-derived dendritic cells (MoDCs). When valine was added to the culture media, it significantly increased the production of IL-12 and enhanced the allo-stimulatory function of human MoDCs. IL-12 is a key cytokine involved in the differentiation of naïve T cells into Th1 cells, which play a crucial role in the immune response. Elevated levels of valine may help modulate the Th1/Th2 balance, potentially influencing the overall immune response [5]. However, there is currently no published works in the literature on the response of canine cells to valine supplementation.

Leucine plays a crucial role not only in protein synthesis but also in cellular metabolism and signal transduction, making it essential for maintaining dogs’ mobility and activity. By regulating the mTOR signaling pathway, leucine significantly influences T cell activation and function [6,7]. Beyond its effects on T cells, leucine’s impact on B cells has been explored. In a murine colorectal cancer model, a leucine-rich diet increased the generation of leucyl-tRNA synthetase 2 (LARS2)-expressing regulatory B cells, which contribute to immune evasion in colorectal cancer [8,9]. Leucine consumption prompts significant metabolic reprogramming of B cells to modulate cancer immunity.

Isoleucine plays a role in immune response by incorporating into proteins found in immune cells, such as lymphocytes, eosinophils, and neutrophils [10]. In a rotavirus-infected piglet model, L-isoleucine not only supported animal growth but also enhanced both innate and adaptive immune responses through the activation of pattern recognition receptors (PRR) signaling pathways [11]. Rotavirus (RV) infection is a major cause of diarrhea in infants and young animals. In dogs, it has been shown to have a higher prevalence in puppies under 3 months of age [12]. Further studies are needed to explore the nutritional and immunological benefits of isoleucine, particularly in puppies, as part of BC supplementation.

Methionine in whey protein aids the synthesis of phosphatidylcholine and acetylcholine, while cysteine is a precursor for antioxidants. Both support the immune system via glutathione, which is crucial for immune function and tissue repair [13]. Additionally, methionine assists DNA methylation and protein synthesis, and plays a key role in the development and differentiation of T and B cells [14,15]. Overall, it may influence the epigenetic regulation and activation of immune cells, impacting the immune response.

In addition to BCAAs, aromatic amino acids help regulate immune functions. Tryptophan, a key amino acid in BC, is broken down by gut microbes into indoles, which bind to the aryl hydrocarbon receptor (AhR). AhR, an activated transcription factor, influences Treg development and IL-10 production, which is crucial for adaptive immunity and maintaining tolerance [16]. For B cells, AhR supports proliferation after B cell receptor activation, and its deficiency leads to decreased class switching to IgG3 and IgA, leading to abnormalities [17]. Although direct canine studies are lacking, human and rodent research suggest promising directions for canines. In summary, whey protein has been widely recognized for its ability to enhance anti-inflammatory and anti-infection responses, and immune regulation. It also contributes to building muscle mass, increasing bone mineral density, and improving mobility and activity levels in recipients.

Besides being a high-quality protein, whey protein also contains several key bioactive components, including α-lactalbumin, β-lactoglobulin, lactoferrin, albumins, Igs, cytokines, lysozyme, and lactoperoxidase [18]. Additionally, whey protein is rich in growth factors that contribute to various biological functions, such as colony-stimulating factor, epidermal growth factor, fibroblast growth factor, insulin-like growth factor, and transforming growth factor [2,18]. While these bioactive components are of bovine origin, the cross-species functionality of BC has been well established. These properties make BC a highly functional food ingredient for both nutritional and medical applications in humans and animals [1,18].

##### α-Lactalbumin

Among the whey protein in BC, α-lactalbumin is the second most abundant protein, comprising approximately 15 to 20% of the total protein content. It is rich in essential amino acids, contributing significantly to the nutritive value of BC [19]. α-lactalbumin acts as a coenzyme in lactose biosynthesis and plays a key role in calcium transport [19]. Additionally, this protein possesses antimicrobial and anti-inflammatory properties, further enhancing the immune benefits of BC for both humans and animals [20,21].

##### β-Lactoglobulin

β-lactoglobulin is the most abundant whey protein in BC, making up over half of the total whey protein content [22]. Its amino acid profile provides significant nutritional and functional value, particularly in the food industry. Methionine, found in β-lactoglobulin, supports cellular methylation and DNA stability [14]. β-lactoglobulin also has strong antioxidant and antimicrobial properties, inhibiting enteric pathogen binding and potentially preventing infections [23]. A study by Oevermann et al. showed that chemically modified β-lactoglobulin inhibited HSV-1 replication in vitro [24]. Hydrolysates of α-lactalbumin and β-lactoglobulin (digested with gastrointestinal enzymes) exhibited antimicrobial and antifungal activity against both Gram-positive and Gram-negative bacteria, as well as yeasts. These hydrolysates also enhanced phagocytic activity in mice, suggesting strong immunostimulatory effects [25]. Moreover, β-lactoglobulin inhibits the adhesion of *E. coli* and *Klebsiella oxytoca*, suggesting it could support intestinal health by preventing opportunistic pathogen growth [26].

##### Lactoferrin

Another important bioactive component in whey protein is lactoferrin (LF), a monomeric glycoprotein with iron-binding capacity like transferrin. LF is heat-resistant and stable against proteolytic enzymes [27]. BC contains approximately 0.8 g/L of lactoferrin [28]. LF is released by activated neutrophils during trauma, infection, and inflammation, where it plays multiple roles, including antimicrobial, antiviral, antioxidant, anti-inflammatory, and immune-regulating functions [29]. Additionally, LF has been shown to have anti-allergic effects. In a murine immune contact dermatitis model, LF administration suppressed allergic immune responses and modulated cytokine production [30].

##### Immunoglobulins

Immunoglobulins (Igs) are key components of BC that provide passive immunity to newborn calves by transferring maternal antibodies. Bovine Igs have immunomodulatory, antimicrobial, and anti-inflammatory effects, including preventing pathogens from binding to cells, activating immune cells for cellular and humoral immunity, modulating the gastrointestinal microbiome, and promoting the generation of secretory IgA [31]. Compared to mature milk, BC contains significantly higher levels of Igs (40–200 mg/mL), with IgG1 being the dominant isotype (over 75%), followed by IgM, IgA, and IgG2 [32]. This high concentration of Igs provides an immune advantage, contributing to 70–80% of the total protein in BC, compared to just 1–2% in mature milk [32]. After birth, calves absorb Igs from BC and milk via the small intestine, allowing protective antibodies to enter the bloodstream, thus transferring passive immunity. Igs from BC can also be absorbed into the blood of other species, including human infants, lambs, foals, piglets, and puppies [33,34]. In summary, BC not only provides essential nutrition for growth but also delivers immune components that protect against pathogens, making it a unique and valuable immune-supportive ingredient.

##### Cytokines

Cytokines are regulatory proteins that play a key role in cell signaling and immune activation. Secreted by various cell types in response to signal transduction, they help trigger immune responses by stimulating, activating, and recruiting immune cells, and neutralizing pathogens. Cytokines are essential for both innate and adaptive immunity. The body’s ability to release specific cytokines in response to certain triggers is an important indicator of health status. In BC, significantly higher levels of pro-inflammatory cytokines (e.g., IL-1β, TNF-α, IFN-γ, and IL-6) and anti-inflammatory cytokines (e.g., IL-1RA) have been observed compared to mature milk [35]. These cytokines support immune system development in newborns, enhance innate immune function, promote antibody production, and may modulate neonatal immunity [36]. The immune-modulatory effects of BC extend beyond cytokine production. In a study by Shing CM and colleagues, human peripheral blood cells stimulated with phytohemagglutinin showed significantly increased secretion of IL-10 and IL-2, alongside reduced levels of IFN-γ and TNF-α when exposed to BC [37]. In a murine calf fecal microbiota transplantation (FMT) model, BC administration protected intestinal barrier integrity and reduced inflammation following *S. typhimurium* challenge. Further analysis reveals enhanced IL-10 secretion and decreased IL-6 and IFN-γ production compared to the untreated control group, highlighting BC’s immunomodulatory properties [38].

##### Enzymes, Growth Factors, and Others

BC contains several bioactive enzymes and growth factors, including lactoperoxidase, lysozyme, trypsin inhibitor, insulin-like growth factors (IGF-1 and IGF-2), and transforming growth factor-β (TGF-β), all of which contribute to its biological activity.

Lactoperoxidase, an antimicrobial glycoprotein, is primarily secreted by the mammary and salivary glands. It plays a crucial role in protecting against pathogens in biological fluids like milk and colostrum. As one of the most abundant enzymes in milk, accounting for 1% of whey proteins, lactoperoxidase supports the nonspecific immune response by producing reactive oxygen species that kill bacteria and inhibit pathogen replication through the innate immune pathway [39]. This helps ensure the safety and nutritional value of BC, particularly by inhibiting foodborne pathogens.

Lysozyme, another enzyme in BC, has antibacterial properties, particularly against Gram-negative bacteria, by breaking down cell walls. It can also inhibit Gram-positive bacteria [40]. Studies have shown that lactoferrin (LF) can enhance the ability of lysozyme to kill *E. coli* [41].

Trypsin inhibitor levels in BC are 100 times higher than in mature milk [42]. It helps preserve the functionality of bioactive components like Igs and growth factors in the gastrointestinal tract, preventing degradation and ensuring their absorption [42].

In addition to enzymes, BC contains bioactive peptidic growth factors, like IGF-1 and IGF-2, which promote cell growth, proliferation, and tissue repair [43]. The key immune-regulatory growth factor in BC is TGF-β2, which can either stimulate or inhibit cell growth depending on the context [44]. TGF-β2, accounting for 94% of TGF-β in the whey fraction, varies in concentration from 150 ng/mL to 1140 ng/mL in the first milking [44]. It is believed to regulate intestinal epithelium growth and influence mucosal immune function in neonates [45].

#### 2.1.2. Casein

In BC, caseins make up 20–30% of the protein [18]. Rich in amino acids like histidine, methionine, and phenylalanine, they offer significant nutritional benefits. Caseins function as a key mineral binder, particularly for calcium and phosphorus, enhancing digestion and nutrient absorption. They also protect bioactive peptides, such as epidermal growth factor (EGF) and IGF-1, from enzymatic breakdown [46,47]. Studies have shown caseins support antimicrobial and immune-regulatory activities [48,49] and provide metabolic and protective effects in conditions like infection, diabetes, and malignancy [50]. Overall, caseins are both a rich nutrient source and a contributor to immune health through its antibacterial and anti-inflammatory properties.

### 2.2. Carbohydrates

BC contains various carbohydrates, including lactose, oligosaccharides, and nucleotide sugars. Lactose makes up about 2.5% of the total carbohydrates in BC [51], providing essential energy to newborns [52]. It has shown immune-modulating properties by inhibiting the suppressive function of regulatory T cells (Tregs) in vitro [53]. Additionally, BC’s lower lactose content reduces the risk of lactose intolerance compared to mature milk [54].

Oligosaccharides, comprising approximately 1g/L in BC (double that of mature milk) [55], also contribute to immune health. These indigestible carbohydrates act as prebiotics, promoting beneficial gut bacteria and enhancing intestinal microbiome health [56]. Major oligosaccharides in BC, such as 3′-sialyllactose (3′-SL) and 6′-sialyllactose (6′-SL), support gut homeostasis by generating short-chain fatty acids through bacterial fermentation. This process improves epithelial cell growth, strengthens the intestinal barrier, and helps prevent pathogen adhesion to gut cells [57]. We conducted a study to analyze oligosaccharides in dog milk from different breeds [58]. Milk samples were collected at various stages of lactation from seven dogs. We identified key oligosaccharides, including 3′-SL, 6′-SL, 2′-fucosyllactose (2′-FL), and lactose-3-sulphate; 3′-SL was the most abundant oligosaccharide, ranging from 6.3–11 g/L on day 1, dropping to 1.5 g/L by day 10. In contrast, 6′-SL levels started at 0.3–0.6 g/L on day 1, peaked on day 5, and remained stable throughout lactation. The levels of 2′-FL varied by breeds, with Schnauzers having the highest levels (around 1.2 g/L) during days 2–4, dropping to 0.5 g/L later. These findings highlight the nutritional importance of oligosaccharides in canine milk, like the benefits provided by bovine colostrum (BC) for dogs.

### 2.3. Fat

BC contains about 7% fat, which is higher than that in mature milk. The fat composition is predominantly saturated fats (about two-thirds), followed by monounsaturated (MUFA) and polyunsaturated (PUFA) fats [59]. BC is rich in n-3 and n-6 PUFA, as well as palmitic and myristic acids, compared to mature milk [60]. The most abundant MUFA in BC is oleic acid, constituting around 20% of total fatty acids. Oleic acid is known for its cardiovascular health and anti-inflammatory benefits [61]. Other lipid components, such as short-chain fatty acids, phospholipids, and gangliosides, may also contribute to health benefits [62]. Notably, lipid components from the milk fat globule membrane (MFGM) have been shown to inhibit rotavirus infectivity in vitro [63]. In a clinical trial, phospholipids and sphingolipids from the MFGM were found to positively affect pathogen attachment and colonization, enhancing resistance to *E. coli* infections [64].

### 2.4. Vitamins

BC contains higher levels of vitamins compared to mature milk, offering both nutritional and immunological benefits [2]. Among the fat-soluble vitamins, vitamin D plays a critical role in immune cell differentiation, calcium and phosphorus absorption for bone mineralization, and overall bone and joint health [65]. Another important vitamin in BC is vitamin K, present in two forms: K1 and K2 [66]. While traditionally known for its role in blood coagulation, recent studies have highlighted vitamin K’s immune-regulatory effects, including its benefits on inflammation, autoimmunity, allergic reactions, metabolic disorders, intestinal microbiota, and even anti-aging effect [67].

### 2.5. Minerals

Dairy products, especially BC, are a significant source of calcium, crucial for strong bones and overall health. Compared to mature milk, BC contains substantially higher levels of calcium, along with other essential minerals like zinc, phosphorus, and iron [2]. Calcium is vital for the development and maintenance of bones and teeth, but it also plays a role as a second messenger in immune cells such as lymphocytes, mast cells, and thymocytes. Calcium signaling helps regulate immune cell functions like differentiation, phagocytosis, migration, and cytokine secretion [68].

Phosphorus, the second most abundant mineral in the body, is essential for bone formation, muscle contractions, heart function, nerve signaling, and protein synthesis. It also supports tissue repair and energy metabolism. Additionally, phosphorus promotes bacterial growth, contributing to a healthy microbiome and enhancing the body’s defense against infections [69]. In brief, bovine colostrum (BC), rich in bioactive components, serves as a powerful superfood, providing a natural source of essential nutrients that support immune health. Key nutrients like proteins, lipids, carbohydrates, vitamins, and minerals all work synergistically to boost immune function and overall well-being. A summary of the key information is presented in Table 1.

## 3. Characteristics of Canine Immune System

Throughout a dog’s life, the immune system undergoes dynamic changes to adapt to different physiological stages, ensuring effective surveillance and protection against diseases. Understanding these changes is the key to provide optimal healthcare for our canine companions. According to the 2019 American Animal Hospital Association (AAHA) Canine Life Stage Guidelines, dogs progress through five distinct life stages, each with unique features in their immune system [70]. Table 2 summarizes these stages, including their definitions and key immune characteristics.

For newborn puppies, infections are a leading cause of morbidity and mortality due to their immature immune systems. Unlike human neonates, who receive significant amounts of maternal antibodies via placental transfer in utero, studies suggest that only 5% to 10% of maternal antibodies are transferred to puppies through the endotheliochorial placenta [71]. This limited transfer makes puppies highly vulnerable, especially since more than 40% of puppies with serum IgG levels below 2.3 g/L may not survive the first three weeks of life, compared to those with higher IgG concentrations (*p* = 0.001) [72]. This genetic and physiological vulnerability highlights the critical importance of passive immunity transfer through colostrum for newborn puppies, providing essential nutrients and immune protection. The neonatal stage is particularly vulnerable for puppies, making the availability of proper nutrients essential for their survival and healthy development. Without colostrum, puppies may experience weakness, slow growth, developmental delays, or even death [72]. There are situations where puppies may be deprived of colostrum, such as in orphaned puppies or in large litters, where the first-born pups may consume most of the colostrum, leaving the later-born puppies without it. In these cases, BC can play a crucial role as a functional food, providing important nutritional support and immune provision when mother’s milk is unavailable or insufficient for the puppies.

BC can serve as an alternative source of nutrients, particularly when dog colostrum is unavailable. While BC does not contain antibodies specific to canine pathogens, it still provides a range of common mammalian proteins that can support immune function. A comparison between BC and dog colostrum revealed similar levels of protein (130 g/kg vs. 138 g/kg) and lactose (31 g/kg vs. 27 g/kg), but BC had lower fat content (36 g/kg vs. 78 g/kg) [73]. The immune components in BC, such as α-lactalbumin, β-lactoglobulin, and lactoferrin, offer antimicrobial and antifungal benefits that can help protect the gut by preventing pathogen attachment and colonization. However, the specific immune benefits of BC for newborn puppies will require further clinical trials to assess its efficacy. Puppies’ immune systems take 6 to 12 months to fully mature, with the timeline varying based on breed, genetics, and nutrition [74]. During this critical period, their immune systems are not fully capable of defending against diseases, making proper nutrition essential. This is a key phase for “immuno-nutrition” to help strengthen their immune systems for long-term health. Research by Rossi L et al. has highlighted the benefits of nutritional supplements in milk replacers, including BC, prebiotics, probiotics, and polyunsaturated fatty acids (PUFAs) [75]. Additionally, a study involving twenty Great Dane bitches demonstrated that supplementation with prebiotics and probiotics during gestation improved the serum concentrations of immunoglobulins (IgG, IgM, and IgA) in both the mothers and their puppies, supporting immune development [76].

Aging is an inevitable process that leads to gradual dysfunction in cells, tissues, and organs, including the immune system, which becomes less effective at protecting the body. Dogs are generally considered seniors starting at age seven [77], though the onset of old age can vary based on breed and size. Research on DNA methylation and cross-species comparisons suggests that aging begins at around 7 years for dogs, with an average lifespan of 10–13 years [78]. As dogs enter their senior years, they experience immune system decline, known as “immunosenescence”, which results in greater susceptibility to infections and poorer responses to vaccinations [79].

Immunosenescence refers to the gradual decline in immune system function due to aging. Both innate and adaptive immunity become downregulated, and the structure of lymphoid organs and immune cells undergoes changes, such as thymic involution and alterations in T cell subpopulations (e.g., a reduced CD4/CD8 ratio, loss of naïve T cells, and an increase in memory T cells). These changes make the body more vulnerable to infections, autoimmune diseases, age-related conditions, and cancers [80]. Aging also triggers a chronic low-grade inflammation, a phenomenon known as “inflammaging”, which further contributes to immune system dysfunction [81]. As dogs age, their adaptive immune system undergoes significant deterioration, affecting both cellular and humoral immunity. Studies have shown a decrease in the absolute number of key immune cells, including lymphocytes, monocytes, and granulocytes, in aging dogs such as Labrador Retrievers [82]. One common method to assess cellular immune function is through T cell proliferation assays using mitogens like Con A, PHA, PWM, and SEB. Aging dogs exhibit a marked reduction in T cell proliferation compared to younger dogs [83]. Additionally, in lymphoid organs like lymph nodes, the ratio of naïve to memory T cells shifts, with memory cells becoming dominant [83,84].

Humoral immunity also declines with age. While the memory response of B cells remains intact, older dogs show a compromised primary response to vaccinations, making them more vulnerable to infections [85]. These immune changes lead to a general loss of immune function, explaining why elderly dogs are more susceptible to infections and why vaccines are less effective in older dogs [79].

## 4. Bovine Colostrum for Canine Growth and Immunity

Colostrum is a highly valuable nutrient that enhances resistance to infections and diseases across different species and age groups. BC can be administered on its own or in combination with other treatments, such as antibiotics, to help fight infections. To date, no side effects or medication interactions have been reported with the supplementation of high-quality colostrum, making it an exceptionally safe and effective nutraceutical option. BC has also shown promise in promoting the healing of intestinal lesions and improving nutrient absorption in the digestive system [86,87].

Given the variations in the canine immune system across different stages of growth and development, it is important to consider the specific immune benefits and health implications of BC for dogs at different life stages. Below, we will summarize how BC can support the immune health of puppies, adult dogs, and senior dogs, highlighting its role as a functional food that supports immune resilience and overall health.

Following the neonatal stage, puppies face a period of immune vulnerability during weaning. As passive immunity from colostrum and mother’s milk fades, and semi-solid or solid food becomes their main nutritional source, the immature immune system is challenged by a range of new environmental factors. These may include changes in their living conditions, such as being separated from their mother and siblings, as well as the stress of moving to a new home. Such stresses increase the risk of infections, gastrointestinal disturbances, and other health issues. Diarrhea is especially common in recently weaned puppies, often because of stress or diet changes. A study by Giffard C.J. et al. [34] involving 70 puppies (primarily toy breeds, aged 40–50 days) investigated the effects of BC supplementation in alleviating stress-induced diarrhea. Puppies were given either 0.5g of BC powder or 0.5 g of skim milk powder (placebo) daily for 10 days after experiencing travel stress. While both groups showed improved fecal quality, puppies fed with BC had significantly lower average fecal scores from day 6 to day 9 of the trial (*p* < 0.05), suggesting BC may help reduce stress-related diarrhea and improve survival rates in young puppies [34]. However, immune biomarkers were not included in this study.

In contrast, a 40-week placebo-controlled clinical trial conducted by our team investigated the longer-term effects of BC on the immune health of adult dogs [88]. Twenty-four husky-cross dogs (aged 2–7 years) were randomized into two groups and fed a complete and balanced diet, with or without BC supplementation. Results show that dogs receiving BC supplementation had significantly elevated fecal IgA levels compared to the control group. IgA is the primary antibody found on mucosal surfaces, playing a key role in defending against pathogens [89]. Additionally, the BC group demonstrated increased fecal microbiota diversity and stability, highlighting positive effects on gut health and immune function. Dogs on the BC diet also exhibited improved humoral immunity, as indicated by significantly higher IgG levels after vaccination with the canine distemper virus (CDV) vaccine. Importantly, no side effects were observed, and there were no significant changes in blood markers like C-reactive protein (CRP), suggesting that BC supplementation did not overstimulate the immune system [88]. This long-term study provides strong evidence for the benefits of BC in supporting gut health, enhancing vaccine efficacy, and boosting immune function in dogs, making it a valuable addition to veterinary care for promoting overall health and well-being. Bovine colostrum (BC) has been tested in combination with other functional foods to boost immune function in dogs. A pilot crossover trial conducted by Dequenne M. et al. [90] investigated the effects of BC supplementation (1 g) combined with four probiotic strains (2.9 × 10^9^ CFU) in eight neutered female Beagle dogs (mean age 8 years). The dogs were divided into two groups, with each group receiving either BC and probiotics or a placebo over three weeks. While the study found improvements in protein digestibility in the BC-supplemented group, there were no significant changes in fecal microbiota or measurable immune benefits [90].

## 5. Conclusions

Bovine colostrum (BC), rich in nutrients, immune regulators, and bioactive molecules, offers possible health benefits for dogs at various life stages. Studies have highlighted BC’s immune-modulating properties, supporting overall health and immune function, making it an ingredient of interest in dog food. Continued research of BC in puppy food and supplementary veterinary care to aid in the nutrition of sick dogs will be beneficial to evaluate the contributions of BC in aiding in optimizing health and in the management of disease.

## Figures and Tables

**Table 1:** Summary of constituents of BC involving modulation of the immune function.

Bioactive Components	Effect on Immune System	References
α-lactalbumin	Antimicrobial and anti-inflammatory properties	[20,21]
β-lactoglobulin	Antioxidant, antimicrobial activity, prevent pathogens’ attachment and colonization	[23,24,25,26]
Lactoferrin	Antimicrobial, antiviral, antioxidant, anti-inflammatory, and modulation of immune responses	[29,30]
Immunoglobulins	Passive immunity, build up barrier against pathogens, activate immune cells to initiate adaptive immunity, prevent infections from spreading to other parts and induce the generation of secretory IgA	[32,33,34]
Cytokines	Immune regulation, activation, and recruitment, cellular signaling, and pathogen recognition, adaptive immunity	[36,37,38]
Lactoperoxidase	Antibacterial, antimicrobial that inhibits bacterial metabolism	[39]
Lysozyme	Antibacterial activity through enzymatic degradation of the cell wall component of the bacteria	[40,41]
Trypsin inhibitor	Keeps the functionality of bioactive components from degradation in the gastrointestinal system, and make them available for absorption	[42]
TGF-beta	Immune regulation, mediators of mucosal immunity	[44,45]
Casein	Immune regulation and antibacterial activity	[48,49]
Lactose	Immune modulation	[53]
Oligosaccharides	Promote growth of beneficial intestinal flora, reduce binding of pathogenic microbiota to the gut epithelium, mucosal immunity	[56,57]
Oleic acid	Anti-inflammatory	[61]
Phospholipids	Anti-infective property, affect pathogen attachment and colonization	[64]
Vitamin K	Beneficial effects on inflammation, autoimmunity, allergic reaction, metabolic disorders, intestinal microbiota modulation, and anti-aging	[67]
Calcium	Modulate the survival and physical function of immune cells	[68]
Phosphorus	Support bacteria reproduction and survival, maintain a stable microbial ecosystem providing anti-infectious benefit to the recipients	[69]

**Table 2:** Immune life stages of dogs.

Stages	Definition (Varying with Breed and Size)	Immune Characters
Puppy	Birth to cessation of rapid growth	Passive immunity from colostrum, functional but immature immune systems; onset of immunocompetence, balance Th1/Th2.
Young adulthood	Transition from rapid growth stage to maturation	Immune system development and re-organization
Mature adult	Completion of physical and social maturation	Immunocompetence
Senior	7 and 7+ years	Immunosenescence, impairment of cellular and humoral immunity, reduction in naïve T cells.
End stage	Endpoint of life	

## Data Availability

No new data were created or analyzed in this study.
